# Daptomycin-Induced Acute Eosinophilic Pneumonia

**DOI:** 10.7759/cureus.13509

**Published:** 2021-02-23

**Authors:** Gilda M Portalatin, Jodi-Ann Chin, Brian Foster, Kevin Perry, Carla McWilliams

**Affiliations:** 1 Internal Medicine, Cleveland Clinic Florida, Weston, USA; 2 Internal Medicine, Southeast Health, Dothan, USA; 3 Infectious Disease, Cleveland Clinic Florida, Weston, USA

**Keywords:** shortness of breath, cough, daptomycin, daptomycin-induced pneumonitis, eosinophilic pneumonia, acute eosinophilic pneumonia, pneumonia, daptomycin-induced acute eosinophilic pneumonia, infectious disease, macrolide antibiotics

## Abstract

Antibiotic use in pneumonia is a common practice globally when there is suspicion for bacterial involvement. However, there have been few instances where the treatment is the cause of pulmonary symptoms, manifesting as so-called “multifocal pneumonia.” Daptomycin is one of the main antibiotics known to have several adverse effects, including drug-induced pulmonary eosinophila. We present the case of a patient with probable daptomycin-induced acute eosinophilic pneumonia. Stopping the offending agent and concomitant steroid therapy resulted in resolution of symptoms and prevention of worsening respiratory distress.

## Introduction

Although acute eosinophilic pneumonia (AEP) is not fully understood, to date, we have seen common denominators whenever patients present, including fever, cough, respiratory distress, new infiltrates on imaging, and a new potential offending agent. It has been described as an acute febrile illness presenting with diffuse pulmonary infiltrates and acute respiratory failure characterized by bronchoalveolar lavage (BAL) eosinophilia and prompt clinical improvement after corticosteroid therapy. AEP is associated with exposure to certain antibiotics, inhalants, non-steroidal anti-inflammatory drugs, infection, and, most importantly, tobacco smoke [1].

Daptomycin, a gram-positive, targeted antibiotic, has been associated with reports of pulmonary toxicity, which can lead to severe acute respiratory distress syndrome (ARDS). It is the leading cause of drug-induced eosinophilic pneumonia (EP), but still remains a very rare entity. The exact mechanism of daptomycin toxicity to the lungs remains unknown; however, the drug undergoes conformational change with calcium interaction, which allows binding to the cytoplasmic membrane, increased membrane permeability, and intracellular ion escape [1]. It is hypothesized that daptomycin binds to pulmonary surfactant, resulting in high concentrations of the drug and causing epithelial injury and inflammation. In addition, the pathophysiology is believed to be caused by antigen detection by alveolar macrophages leading to T-helper 2 lymphocytic recruitment with subsequent release of interleukin-5, thus promoting release of eosinophil production and migration to the lungs [2]. The most common presenting symptoms in patients with AEP include dyspnea followed by pulmonary opacities on chest X-ray (CXR) or computed tomography (CT) findings. Although there is no clear consensus, corticosteroids have been shown to exert beneficial effects in patients with AEP through eosinophilic apoptosis [3]. With increasing use of daptomycin over recent years, it has become important for clinicians to recognize this condition early and manage appropriately.

## Case presentation

A 53-year-old male with a history of hypertension, prediabetes, hyperlipidemia, and ulcerative colitis on mesalamine presented with a non-productive cough, dyspnea, and fever (102°F). Approximately one month prior, he was admitted to another hospital for fever (103°F) and neck pain that started after returning from a Mediterranean cruise. Once admitted to the outside hospital, magnetic resonance imaging (MRI) of the neck showed multiple small cervical paraspinous abscesses. There were no respiratory concerns throughout hospitalization. In addition, he was found to have Methicillin-sensitive *Staphylococcus aureus *(MSSA) bacteremia. Transesophageal echocardiogram was performed and was found to be unremarkable. An infectious disease specialist was consulted, and despite multiple antibiotics, the patient only responded to daptomycin. Thereafter, the white blood cell count remained within normal range (3,800-10,800/µL) and the patient defervesced. He was later discharged on a six-week course of daptomycin via peripherally inserted central venous catheter (PICC) line.

**Figure 1:**
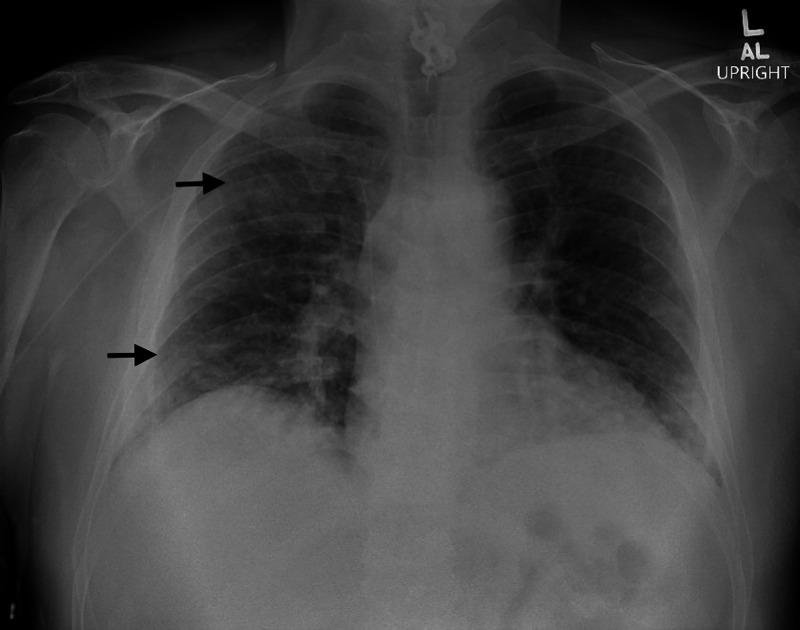
Portable CXR demonstrating multiple opacities concentrated in the right upper and lower lobes (black arrows). CXR, chest X-ray

The patient stated that he was doing well after hospital discharge until one day prior to admission. He began to have recurrent fevers, non-productive cough, and shortness of breath and later presented to the our hospital for further evaluation. Symptoms began 10 days after initiation of daptomycin. In the emergency room (ER), he was febrile to 103.1°F, tachycardic, and hypoxemic with oxygen saturation of 89%, and was subsequently placed on 2 L nasal cannula. His lung examination was significant for crackles, predominately, right upper and lower lung fields and upper extremity examination demonstrated a right-sided PICC with no signs of erythema or tenderness at the site. Laboratory findings were significant for leukocytosis of 18,120/µL (3,800-10,800/µL) and peripheral absolute eosinophilia of 790/µL (15-500 cells/µL). He was evaluated with a CXR which demonstrated patchy areas of ground glass density in the right upper and lower lobes, consistent with lung volume and/or interstitial disease (Figure 1).

He was restarted on daptomycin in addition to piperacillin/tazobactam for possible hospital-acquired pneumonia and recent cervical paraspinous abscesses.

**Figure 2:**
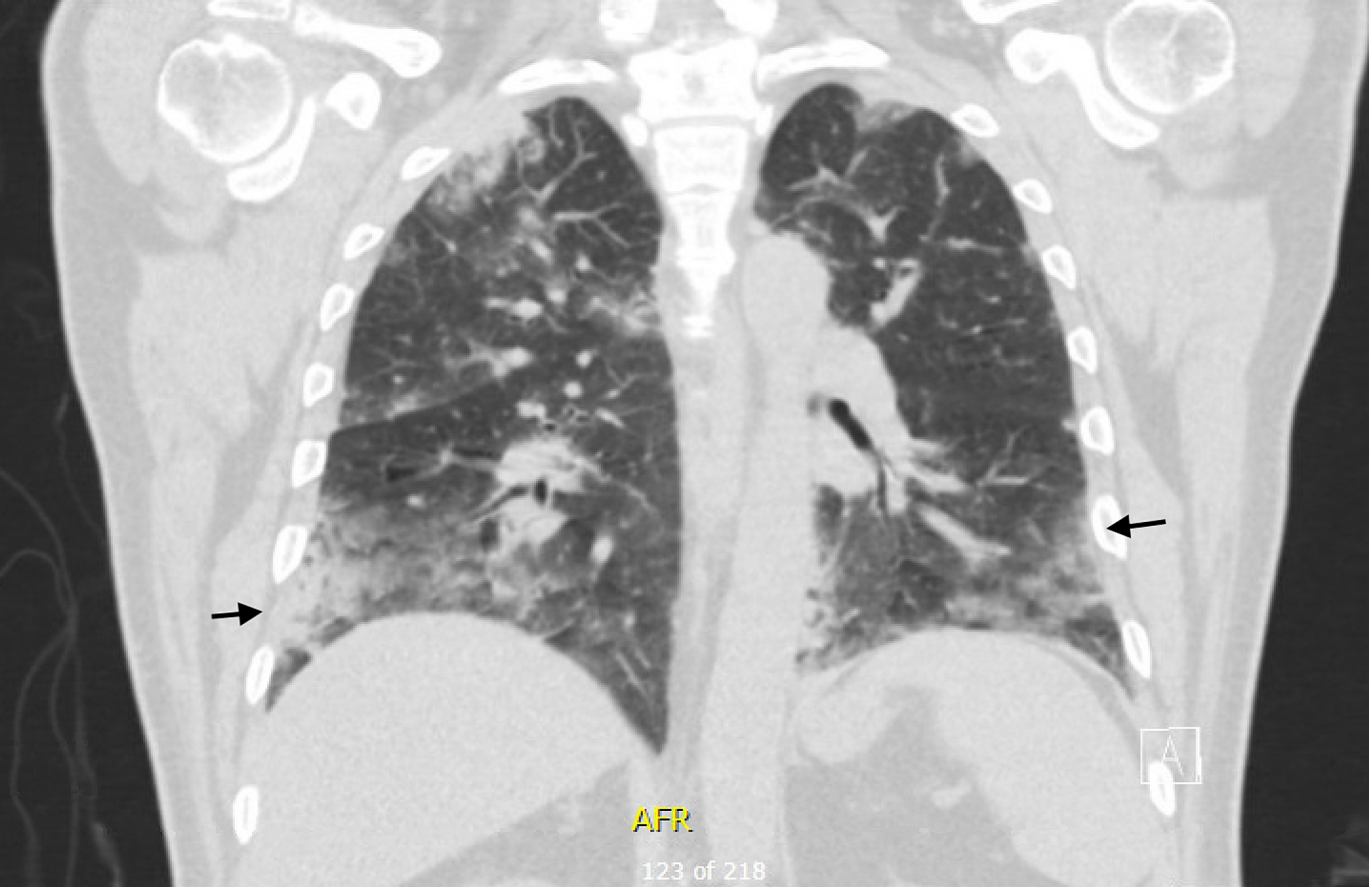
Coronal view of CT chest with diffuse peripheral ground glass, consolidative, nodular opacities in all lobes consistent with multifocal pneumonia (black arrows). CT, computed tomography

**Figure 3:**
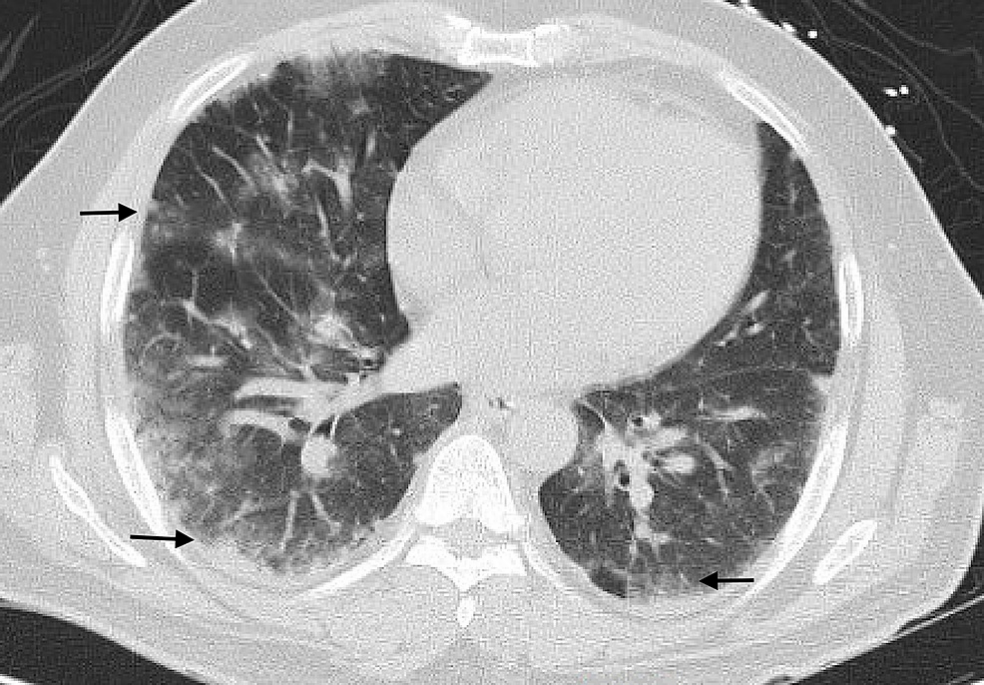
Cross-sectional view of CT chest showing bilateral involvement of ground glass opacities predominately in the periphery (black arrows). CT, computed tomography

The next day, the patient continued to have fevers with persistent cough. Follow-up CT chest showed evidence of diffuse peripheral ground glass, consolidative, nodular opacities in all lobes with evidence of multifocal pneumonia, as noted in the coronal view (Figure 2) as well as the cross-sectional view (Figure 3).

 

Despite broad-spectrum antibiotics, no clinical improvement was noted. Our infectious disease specialist was consulted, and considering high clinical suspicion of daptomycin as the culprit of his symptoms, it was promptly discontinued and he was started on intravenous solumedrol 40 mg daily. Repeat CT and MRI of cervical spine were negative for abscess or discitis; however, given recent MSSA bacteremia and paraspinous abscesses diagnosed at the outside hospital, patient was discharged on intravenous cefazolin for a total of six weeks.

For the next 48 hours of hospitalization, the patient remained afebrile with no requirement of supplemental oxygen while maintaining adequate saturation. At the time of discharge, white blood cell count was down to 9,060/µL and absolute peripheral eosinophil count was negligible. Given clinical improvement with drug cessation, resolution of eosinophilia, a diagnosis of daptomycin-induced EP was made. He was discharged on the following steroid taper: prednisone 30 mg daily for three days, followed by 10 mg every three days for two weeks and 5 mg daily for the final three days. He was scheduled to see Pulmonology for a follow-up visit. BAL was never performed. Repeat chest radiograph four weeks after discharge revealed complete resolution of bilateral opacities, and his pulmonary symptoms had subsided.

## Discussion

Daptomycin-induced AEP is a rare and poorly understood disorder characterized by pulmonary eosinophilia with rapid onset of non-specific symptoms, including dyspnea, fever, and cough that may be mistaken for infectious multifocal pneumonia. Based on the literature review, AEP has a male preponderance and appears to be dependent on time of exposure rather than on the dose. Peripheral eosinophilia may not be present initially in acute EP and increases over time as the disease progresses. Our patient met the following criteria for the probable case of daptomycin-induced AEP: concurrent exposure to daptomycin, dyspnea with increased oxygen requirement, new infiltrates on chest imaging, peripheral eosinophilia, and clinical improvement following daptomycin withdrawal [3]. With only 35 cases identified as true daptomycin-induced AEP, the exact mechanism is unknown. Two proposed theories include daptomycin accumulation near the epithelial alveolar surface causing epithelial injury and pneumonia, and daptomycin interaction with surfactant altering lipid integrity and stimulating an inflammatory response [4].

This case report highlights the importance of recognizing medications and/or chemicals that may precipitate AEP when there is no improvement of pulmonary symptoms. In cases of respiratory failure, BAL may reveal the presence of numerous eosinophils on cytology staining. However, neither BAL nor peripheral eosinophilia is a requirement for diagnosis; our patient had elevated eosinophils along with new infiltrates on CXR/CT and showed improvement of symptoms with withdrawal of daptomycin, which is considered a probable diagnosis of AEP [4]. Corticosteroids have been the mainstay treatment for many AEP cases as they are known to reduce inflammatory states. Literature has shown improvement or even resolution of symptoms within 48-72 hours with prompt initiation of steroids [1]. There is no evidence of whether a longer versus shorter course of steroids has better outcomes. In our patient, we prescribed a two-week course of prednisone with no relapse when evaluated at his four-week follow-up visit. Although prognosis is excellent with drug discontinuation, early detection is key to prevent rapidly progressive respiratory failure and ARDS. Considering the multifactorial causes of AEP and increased prevalence in the last several years, there needs to be much more awareness of these cases of “unresolved pneumonia.”

## Conclusions

While daptomycin-induced AEP appears to be a rare adverse effect of daptomycin use, it can result in severe respiratory failure and resultant morbidity/mortality. Thus, in any patient presenting with respiratory complaints found to be concomitantly on daptomycin, this diagnosis should be considered. It is important to recognize that daptomycin-induced AEP is not related to drug dose, nor is the timing of presentation consistent. Furthermore, quick recognition is imperative as a short course of steroids can treat and reverse impending respiratory failure. Finally, it is noted that further research is needed surrounding the mechanism of daptomycin-induced AEP along with investigations to highlight the long-term sequelae/prognosis of this unfortunate adverse effect.
